# Sugar and Chromosome Stability: Clastogenic Effects of Sugars in Vitamin B6-Deficient Cells

**DOI:** 10.1371/journal.pgen.1004199

**Published:** 2014-03-20

**Authors:** Antonio Marzio, Chiara Merigliano, Maurizio Gatti, Fiammetta Vernì

**Affiliations:** Istituto Pasteur-Fondazione Cenci Bolognetti and Istituto di Biologia e Patologia Molecolari (IBPM) del CNR, Dipartimento di Biologia e Biotecnologie “C. Darwin”, Sapienza, Università di Roma, Roma, Italy; Stowers Institute for Medical Research, United States of America

## Abstract

Pyridoxal 5′-phosphate (PLP), the active form of vitamin B6, has been implicated in preventing human pathologies, such as diabetes and cancer. However, the mechanisms underlying the beneficial effects of PLP are still unclear. Using *Drosophila* as a model system, we show that PLP deficiency, caused either by mutations in the pyridoxal kinase-coding gene *(dPdxk)* or by vitamin B6 antagonists, results in chromosome aberrations (CABs). The CAB frequency in PLP-depleted cells was strongly enhanced by sucrose, glucose or fructose treatments, and *dPdxk* mutant cells consistently displayed higher glucose contents than their wild type counterparts, an effect that is at least in part a consequence of an acquired insulin resistance. Together, our results indicate that a high intracellular level of glucose has a dramatic clastogenic effect if combined with PLP deficiency. This is likely due to an elevated level of Advanced Glycation End-products (AGE) formation. Treatment of *dPdxk* mutant cells with α-lipoic acid (ALA) lowered both AGE formation and CAB frequency, suggesting a possible AGE-CAB cause-effect relationship. The clastogenic effect of glucose in PLP-depleted cells is evolutionarily conserved. RNAi-mediated silencing of *PDXK* in human cells or treatments with PLP inhibitors resulted in chromosome breakage, which was potentiated by glucose and reduced by ALA. These results suggest that patients with concomitant hyperglycemia and vitamin B6 deficiency may suffer chromosome damage. This might impact cancer risk, as CABs are a well-known tumorigenic factor.

## Author Summary

We show that the active form of vitamin B6 (Pyridoxal 5′-phosphate, PLP) plays an important role in the maintenance of genome integrity. We found, using *Drosophila* as a model system, that PLP deficiency results in chromosome breaks and rearrangements (collectively dubbed chromosome aberrations, abbreviated with CABs). Most importantly, we observed that in PLP deficient cells, sucrose, glucose, or fructose strongly enhance the frequency of CABs. The mutagenic effects of sugars in the presence of PLP deficiency are evolutionarily conserved, as PLP depletion or inhibition in human cells results in CAB formation, which is potentiated by glucose or fructose. These results suggest that patients with concomitant hyperglycemic crises and vitamin B6 deficiency may suffer genetic damage, which might promote cancer and diabetes complications. Our work further suggests that patients treated with PLP antagonist drugs should keep under control the level of sugar in their blood and compensate their vitamin B6 level.

## Introduction

It is now widely accepted that chromosome aberrations (CABs) can contribute to cancer development. Deletions, duplications and chromosome exchanges such as dicentrics and translocations can ultimately result in loss of genetic material (loss of heterozygosity), DNA amplification and formation of aberrant gene fusions, thus promoting carcinogenesis [1]–[3]. Tumor development has been also associated with chromothripsis, a phenomenon of massive DNA fragmentation followed by multiple chromosomal rearrangements involving between one and a dozen of chromosomes [4]–[6]. It is currently unclear whether cells with chromothripsis are generated by a single event or result from multiple successive events involving more than one cell cycle [7]–[9].

Abundant evidence indicates that CABs are mainly generated by unrepaired or improperly repaired double strand breaks (DSBs). DBSs can be induced by external agents such as ionizing radiations and chemical mutagens or by endogenous factors such as the free radicals generated by the oxidative metabolism or errors in DNA replication [10]–[13]. DSBs are repaired through two distinct but interconnected mechanisms - non-homologous end joining (NHEJ) and homologous recombination (HR)- both of which are mediated by evolutionarily conserved proteins. NHEJ joins broken chromosome ends directly and relies on the activities of the Mre11-Rad50-Nbs (MRN) complex, the Ku heterodimer, and the Ligase 4 complex. HR and its variant single strand annealing (SSA) are based on recombination with homologous genomic sequences, and exploit a variety of factors including the MRN complex, RAD51, BRCA1, BRCA2, BLM and ATM [10]. Mutations in *ATM* (Ataxia Telangiectasia Mutated), *MRE11*, *NBS1* (Nijmegen Breakage Syndrome), *BRCA1* (Breast Cancer 1), *BRCA2* and *Ligase 4* cause human syndromes characterized by both CABs and cancer predisposition, highlighting the connection between CABs and cancer [14], [15].

Several studies have shown that inadequate intake of micronutrients results in DNA damage and cancer in humans [16], [17]. A micronutrient that protects from DNA damage and is beneficial for cancer prevention is Pyridoxal 5′-phosphate (PLP) [16], [18]–[20]. PLP is the metabolically active form of vitamin B6 generated by pyridoxal kinase; it acts as a cofactor for more than 140 enzymes, which catalyze a myriad of biochemical reactions. It has been estimated that PLP is involved in 4% of all catalytic activities and it is known to play essential roles in wide range of metabolic and developmental processes including amino acid, fatty acid and neurotransmitter metabolism [19]–[21]. There is also evidence that PLP quenches the oxygen reactive species acting as a potent antioxidant [22]–[24] and antagonizes Advanced Glycation End-products (AGE) formation [19], [25], [26].

Based on its wide range of functions it is not surprising that PLP is beneficial for many human diseases. Indeed, many epidemiological studies indicate that PLP protects from cancer, diabetes, cardiovascular diseases and neurological disorders [19], [20]. However, the mechanisms underlying the effects of PLP at the molecular and cellular levels are still poorly understood. Here we show that in both *Drosophila* and human cells an elevated intracellular level of glucose has a dramatic clastogenic effect if combined with PLP deficiency; some cells exhibit an extensive chromosome damage that is reminiscent of chromothripsis. In addition, we show that PLP deficiency greatly potentiates AGE formation. Our findings suggest vitamin B6 deficiency coupled with hyperglycemia results in chromosome damage, which might promote carcinogenesis.

## Results

### Mutations in the *dPdxk* gene cause chromosome aberrations

**Figure 1:**
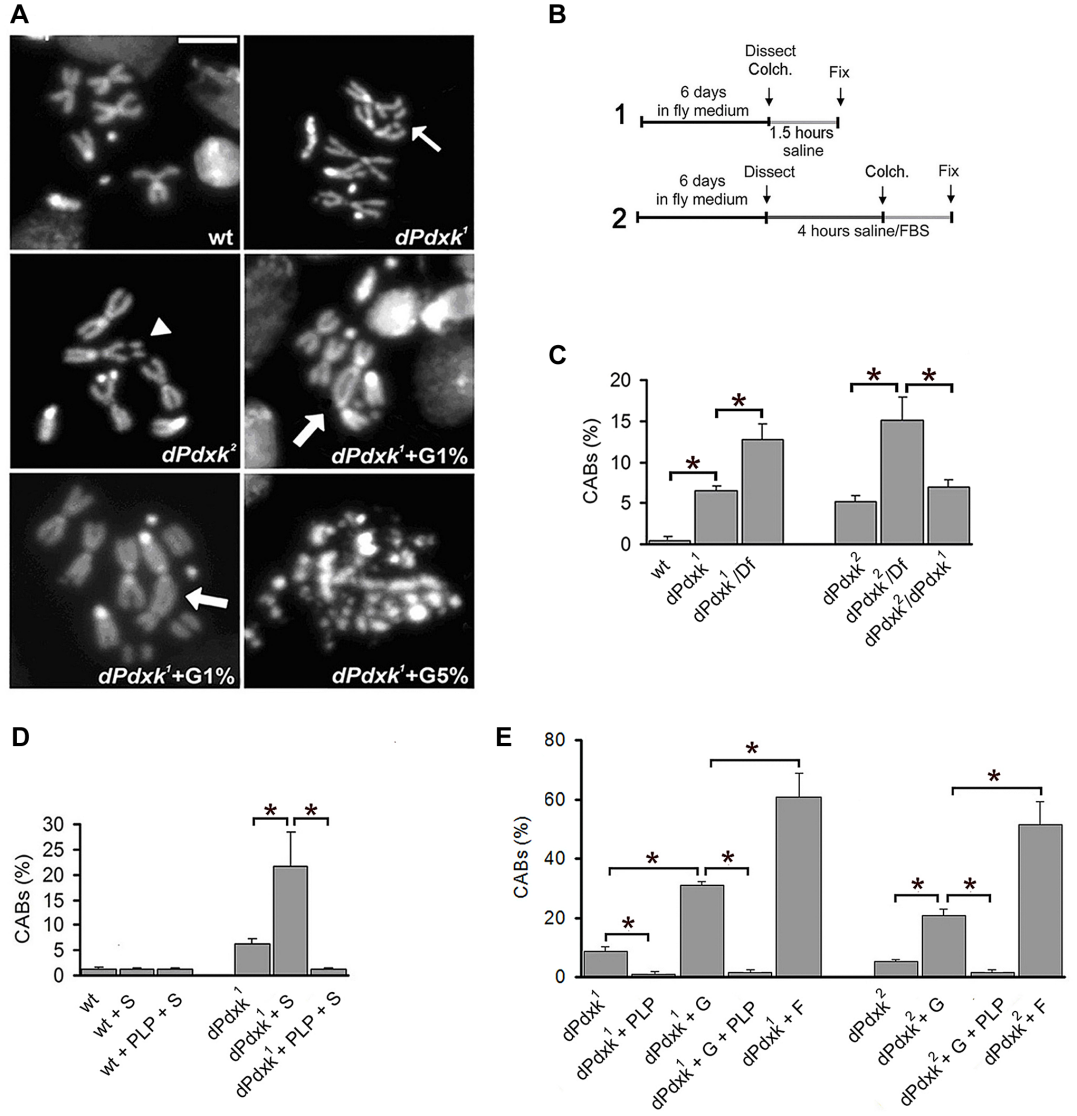
Clastogenic effects of sugar in PLP-deficient *Drosophila* brain cells. (A) Examples of CABs; the thin arrow and the arrowhead point to a chromatid and an isochromatid deletion, respectively; the thick arrows point to multicentric chromosomes. The *dPdxk* mutant cell treated with 5% glucose (G) exhibits extensive chromosome fragmentation. Scale bar, 5 µm. (B) Scheme of the 2 experimental protocols used. Colch, colchicine addition; Fix, fixation. (C) CAB frequencies in wild type (wt) and *dPdxk* mutants (protocol 1); *Df* is *Df(3L)AC1* that uncovers *dPdxk*; (D) CAB frequencies in wt and *dPdxk* mutant brains from larvae grown in normal medium and treated with either sucrose (S) or S plus PLP (protocol 1). (E) CAB frequencies in wt and *dPdxk* mutant brains incubated in saline containing either 1% G or 1% fructose (F) with or without PLP (1 mM) (protocol 2). Each bar of the C, D and E graphs represents the mean frequency of CABs (±SE) obtained by examining at least 800 metaphases from at least 8 brains. *, significantly different in the Student t test with p<0.001.

We identified a mutation (*dPdxk^1^*) in the *Drosophila* gene encoding pyridoxal kinase (Pdxk) by a cytological screen of 1680 third chromosome lines bearing recessive mutations that cause death at late larval stages (see Materials and Methods). Pdxk plays a critical role in the formation of pyridoxal 5′-phosphate (PLP), the active form of vitamin B6 [19]. Mitotic cells from colchicine-treated *dPdxk^1^* mutant brain displayed ∼6% chromosome aberrations (CABs); the frequency of aberrations in wild type cells is ∼0.5% (Fig. 1A–C). Genetic analyses placed *dPdxk^1^* in the 67A9-67B2 polytene chromosome interval that contains only 11 genes (figure S1A). A mutation in one of these genes, *l(3)67Ab*, failed to complement *dPdxk^1^* and has been thus renamed *dPdxk^2^*. The frequencies of CABs observed in *dPdxk^1^/Df(3L)AC1* (67A2-67D11) and *dPdxk^2^/Df(3L)AC1* hemizygotes were significantly higher than those seen in the respective *dPdxk^1^* and *dPdxk^2^* homozygotes (Figures 1C and S1A), suggesting that both mutant alleles are hypomorphic.

DNA sequencing showed that both *dPdxk^1^* and *dPdxk^2^* carry lesions in the Pdxk-encoding *CG34455* gene, which specifies a 304 aa protein (http://flybase.org). *dPdxk^1^* carries an A→G transition (# 338) in a splicing acceptor site, which is predicted to lead to a truncated protein of 83 aa; *dPdxk^2^* carries a T→C transition (# 700) that leads to a phenylalanine to serine substitution in the active site of Pdxk. To confirm the identity of the *dPdxk* gene we performed complementation tests with three different transgenes: one contained the endogenous promoter and the *CG34455* genomic sequence fused in frame with the GFP sequence; the other transgenes were both placed next to the tubulin promoter and contained either the *CG34455* or the human *PDXK* cDNA fused in frame with the 3HA sequence (Figure S1B). All transgenes rescued the CAB phenotype of *dPdxk* mutants; the transgene placed under the control of the endogenous promoter also rescued the *dPdxk^1^* lethal phenotype.

### Sugars increase the CAB frequency in *dPdxk* mutants

**Figure 2:**
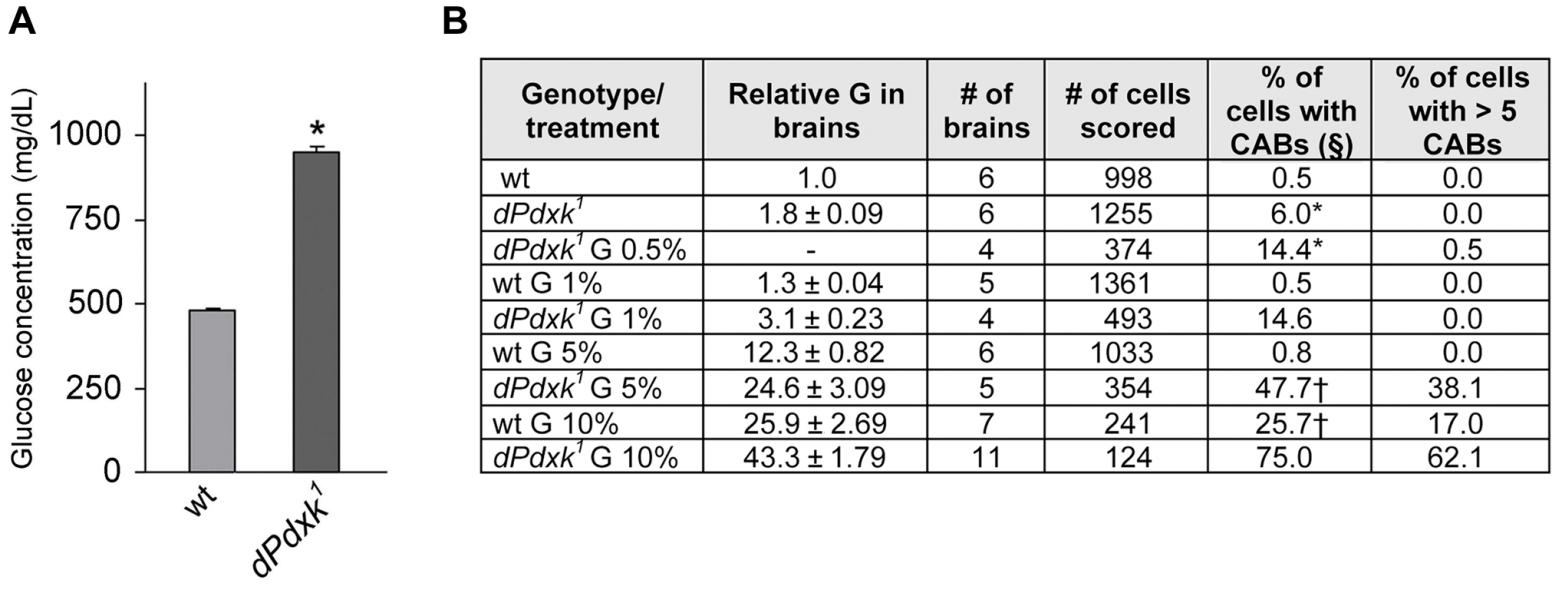
Relationships between PLP, glucose and CABs. (A) Glucose concentration in the hemolymph of wild type (wt) and *dPdxk* mutant larvae. Columns are the means of 8 independent samples of 10 larvae ±SE; *, glucose content is significantly higher than in wild type with p<0.001 in the Student t test. (B) CAB frequencies and relative intracellular glucose (G) concentration in wild type (wt) and *dPdxk* mutant brains incubated in saline/FBS with the indicated G contents (protocol 2; figure 1). §, includes cells with >5 CABs. * and †, significantly different in the Student t test with p<0.001 and p<0.05, respectively.

To ask whether the CAB phenotype was due to PLP deficiency, we grew *dPdxk* mutant flies in food supplemented with 10^−2^ M PLP in 4% sucrose, or in a food supplemented with 4% sucrose only (protocol 1, Figure 1B). The PLP-containing food completely suppressed the CAB phenotype. However, to our surprise, *dPdxk* mutant larvae grown in the food with only sucrose displayed a 3-fold increase in CAB frequency compared to mutant larvae grown in normal food (Figure 1D). Because larval feeding does not allow precise control of sugar and PLP intake, we incubated dissected brains from third instar larvae for 4 h in saline/FBS (0.7% NaCl supplemented with 10% fetal bovine serum) containing defined quantities of sugars or drugs (protocol 2, Figure 1B). This analysis revealed that 1% glucose causes substantial increases in the CAB frequency in both *dPdxk^1^* (3.5-fold) and *dPdxk^2^* (4.1-fold) mutant brains (Figure 1E). The effects of 1% fructose were even more dramatic, as it caused 6.9 and 10.3-fold increases in the CAB frequency in *dPdxk^1^* and *dPdxk^2^* brains, respectively. 1% glucose or fructose did not induce CABs in wild type brains (see Figure 2B below and data not shown). Most importantly, the clastogenic effects of sugars were drastically reduced by 1 mM PLP (Figure 1E).

In addition to glucose and glycogen *Drosophila* cells and hemolymph contain trehalose, a disaccharide formed from two glucose moieties. Trehalose is a particularly stable high-energy storage molecule that can be transported and accumulated to high concentrations without toxic effects [27]. We thus focused on glucose and measured its concentration in *dPdxk* mutant tissues. Using a Hexokinase-based detection method, we found that the hemolymph of *dPdxk^1^* larvae contains nearly twice as much glucose as that of wild type larvae (Figure 2A). We next analyzed the dose-effect relationships between the intracellular glucose concentration (IGC) in larval brain cells and the CAB frequency. Dissected brains were incubated for 4 h in saline/FBS with increasing glucose concentrations and then examined for both the IGC and the presence of CABs (protocol 2, Figure 1B). In *dPdxk^1^* brains exposed at high glucose concentrations the frequency of cells with more than 5 CABs was quite high (Figure 2B) and in many cases the CAB number per cell could not be assessed. Thus, in order to render the data comparable, we considered the frequency of cells with CABs instead of the CAB frequency (number of CABs/number of cells scored) as in Figure 1C–E. In *dPdxk^1^* mutant brains, the frequency of cells with CABs increased with the IGC (Figure 2B). In wild type brains treated with 1% or 5% glucose, the frequencies of cells with broken chromosomes were comparable to that of non-treated controls, and only treatments with 10% glucose resulted in a significant CAB increase (Figure 2B). Importantly, wild type and *dPdxk^1^* brains with comparable IGCs (wt brains in 10% glucose and *dPdxk^1^* brains in 5% glucose) displayed significantly different frequencies of cells with CABs, with mutant brains showing a higher frequency of metaphases with damaged chromosomes than controls (Figure 2B). These results indicate that CABs are not caused by a high IGC only, but by the simultaneous occurrence of an elevated IGC and a low PLP level. Thus, in the presence of low PLP levels (as shown in later figure), sucrose, glucose and fructose behave as potent clastogens.

Interestingly, in brains with high IGC approximately 10% of the metaphases with more than 5 CABs showed an extensive chromosome fragmentation (Figure 1A). The frequency of chromosome breaks and rearrangements in the latter cells is clearly much higher than that expected from a Poisson distribution of CABs. The occurrence of this massive chromosome damage might reflect an increase of IGC beyond a critical threshold in cells where PLP is strongly reduced.

### The spindles of glucose-treated *dPdxk* mutant brains are resistant to colchicine

**Figure 3:**
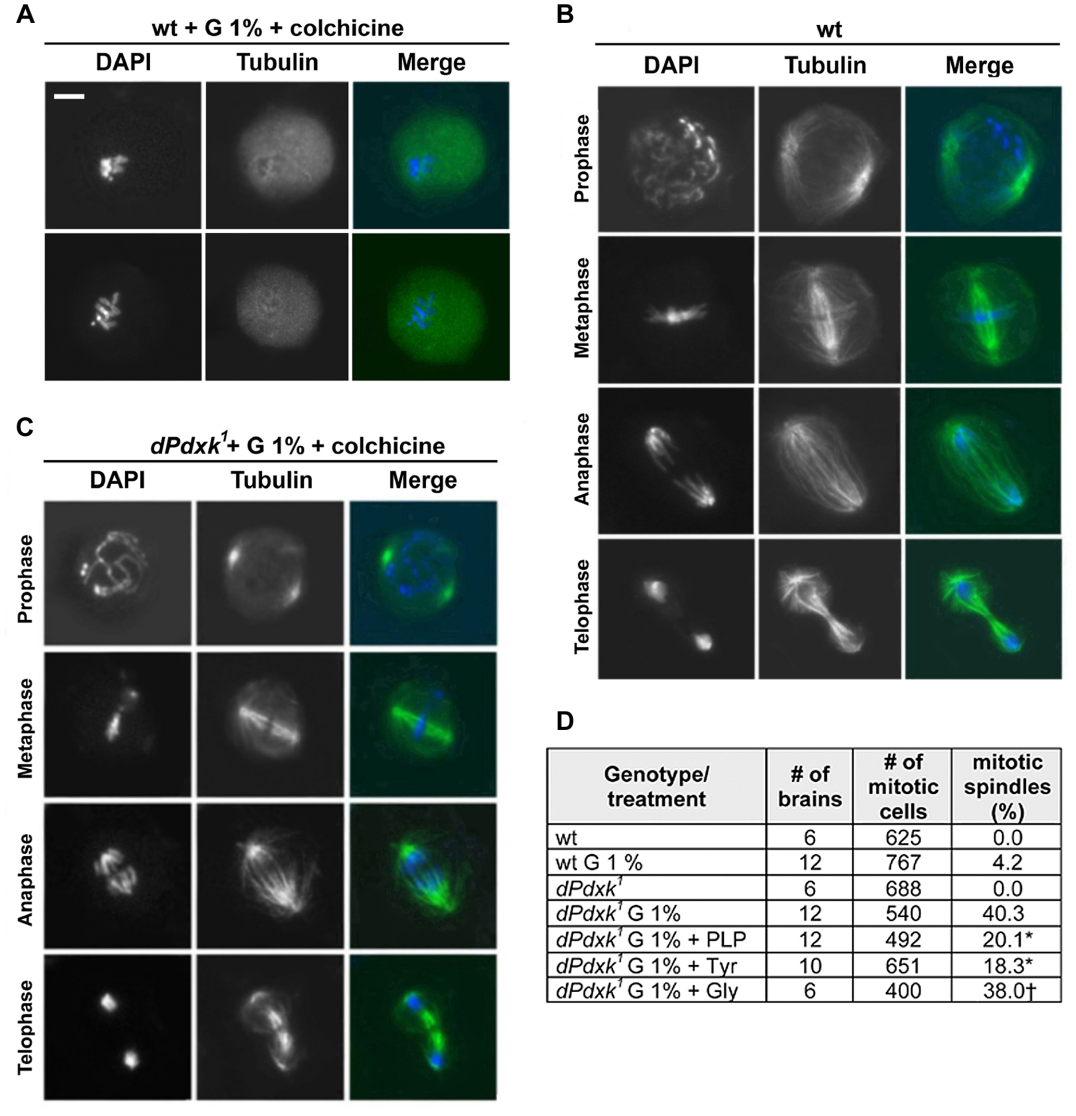
The spindles of glucose-treated *dPdxk* mutant brains are resistant to colchicine-induced depolymerization. (A–C) Cells were immunostained for tubulin (green) and DNA (blue). (A) Examples of metaphase figures from colchicine-treated wild type brains with completely depolymerized spindles. (B) Examples of mitotic spindles of wild type brain neuroblasts. (C) Examples of colchicine-resistant spindles observed in *dPdxk* mutant brains exposed to 1% glucose (G). These spindles exhibit a slightly reduced microtubule (MT) density but are otherwise normal. Scale Bar, 5 µm. (D) PLP (1 mM), Tyrosine (1.7 mM) but not glycine (1.7 mM) partially restores colchicine sensitivity of glucose-treated *dPdxk* spindles. All cells have been treated with 10^−5^ M colchicine for 90 minutes. “Number of mitotic cells” is the total number of mitotic figures that exhibit DAPI-stained chromosomes; “mitotic spindles (%)” is the frequency of mitotic cells that exhibit a colchicine-resistant spindle. Significantly different (*) with p<0.01 or not different (†) from *dPdxk* mutant brains incubated in 1% G only (Student t test).

Additional evidence for a high IGC in *dPdxk^1^* mutant brains was provided by the analysis of mitosis in larval brains. We noticed that preparations of *dPdxk^1^* mutant brains incubated in 1% glucose (protocol 2, Figure 1B), and treated for 90 min with 10^−5^ M colchicine before fixation, contain several anaphases. Immunostaining for tubulin revealed that these preparations display many mitotic spindles with a slightly reduced microtubule (MT) density but otherwise normal (Figure 3A–C). In contrast, preparations of colchicine-treated wild type or *dPdxk^1^* mutant brains did not exhibit any recognizable spindle structure (Figure 3A). We thus asked whether PLP addition alleviates the colchicine resistance of the *dPdxk^1^* mutant spindles incubated in 1% glucose. We found that treatments with 1% glucose (protocol 2, Figure 1B) result in 40.3 % colchicine-resistant spindles. However, if brains were incubated in both 1% glucose and 1 mM PLP, the frequency of resistant spindles was only 20.1% (Figure 3D). These observations suggest that addition of exogenous PLP compensates the PLP deficiency caused by the *dPdxk^1^* mutation, reducing the glucose level and its effects on spindle MTs.

Tubulin tyrosination and detyrosination is a well-known reversible enzyme-mediated post-translational modification that affects MT stability. MTs that end with a tyrosine residue at the C-terminus of α-tubulin (Tyr-MTs) are more dynamic and more resistant to nocodazole-induced depolymerization than detyrosinated MTs (usually called Glu-MTs because their C-terminal residue is Glu instead of Tyr) [28], [29]. Early studies showed that human umbilical vein endothelial cells display colchicine-resistant MTs and reduced proliferation when cultured in high glucose. The MTs of these cells were characterized by frequent loss of the terminal tyrosine residue and their colchicine resistance was corrected by tyrosine addition [30], [31]. Based on these results, we incubated for 4 hours *dPdxk^1^* mutant brains in saline/FBS containing 1.7 mM tyrosine and 10^−5^ M colchicine (added 90 min before fixation). These brains showed a substantial reduction in the frequency of mitotic figures with undepolymerized spindles compared to brains treated in the same way but without tyrosine addition (18.3 *vs* 40.3 %); addition of glycine (1.7 mM) instead of tyrosine did not affect the frequency of colchicine-resistant spindles (Figure 3D). Thus, in line with the studies on human umbilical vein endothelial cells, addition of tyrosine corrects colchicine resistance also in *Drosophila dPdxk* mutant cells that accumulate an elevated glucose amount.

We also studied the effect of tyrosine on CAB formation. We examined the CAB frequencies in *dPdxk* mutant brains incubated in either 1% glucose and 1.7 mM tyrosine or in 1% glucose only (protocol 2, Figure 1B). We found that the frequency of CABs in tyrosine treated brains (18.0 %; 8 brains; 506 metaphases) is not significantly different from that of brains exposed to glucose only (20.9 %; 8 brains; 367 metaphases). These results suggest that colchicine-resistant spindles and CABs are unrelated outcomes of PLP deficiency, as tyrosine affects spindle resistance but not CAB formation.

### PLP inhibitors mimic the effects of *dPdxk* mutations

**Figure 4:**
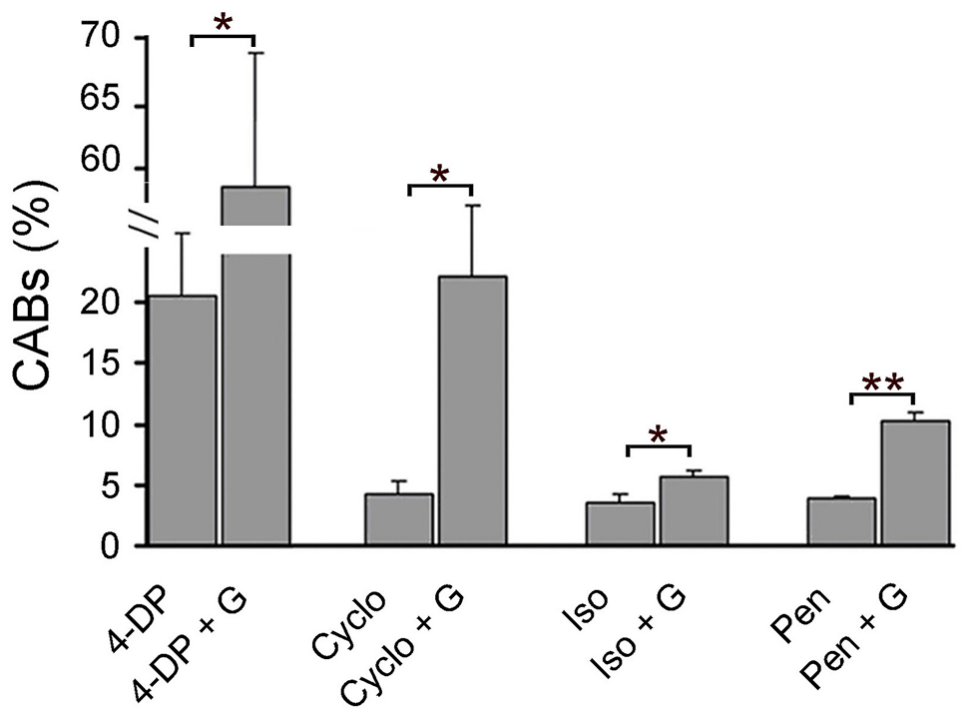
PLP inhibitors cause CABs and their clastogenic effect is potentiated by glucose. CAB frequencies in wild type brains treated with 4-DP (10 mM), isoniazid (Iso, 10 mM) penicillamine (Pen, 10 mM) in the presence or absence of 1% glucose (G) (protocol 2, figure1B). In the cycloserine (Cyclo) experiment, larvae were grown for 6 days in food containing 10 mM cycloserine; dissected brains were then incubated for 4 hours in saline/FBS with or without 1% G. Each bar in the graphs represents the mean frequency of CABs (±SE) obtained by examining at least 800 metaphases from at least 8 brains. * and **, indicate significant difference with p<0.05 and p<0.001 in the Student t test. Untreated wild type brains exhibit 0.5% CABs (see Figure 1).

There are several well-known PLP inhibitors, some of which are used in pharmaceutical treatments. These drugs include the vitamin B6 analog 4-deoxypyridoxine (4-DP), isoniazid (tuberculosis treatment, antidepressant), penicillamine (antirheumatic), and cycloserine (tuberculosis treatment, antidepressant) [32], [33]. Brains from wild type larvae incubated for 4 hours in the presence of 4-DP, isoniazid or penicillamine (protocol 2, Figure 1B) showed higher levels of CABs than untreated controls (Figure 4). When these brains were also exposed to 1% glucose, the CAB frequency was further and significantly increased (Figure 4). Similarly, brains from larvae grown in the presence of cycloserine displayed a significant increase in CABs compared to untreated controls, and this effect was potentiated by glucose addition (Figure 4). These results indicate that glucose induces CABs when vitamin B6 activity is reduced by drug treatments.

### *dPdxk* mutants are partially defective in insulin signaling

**Figure 5:**
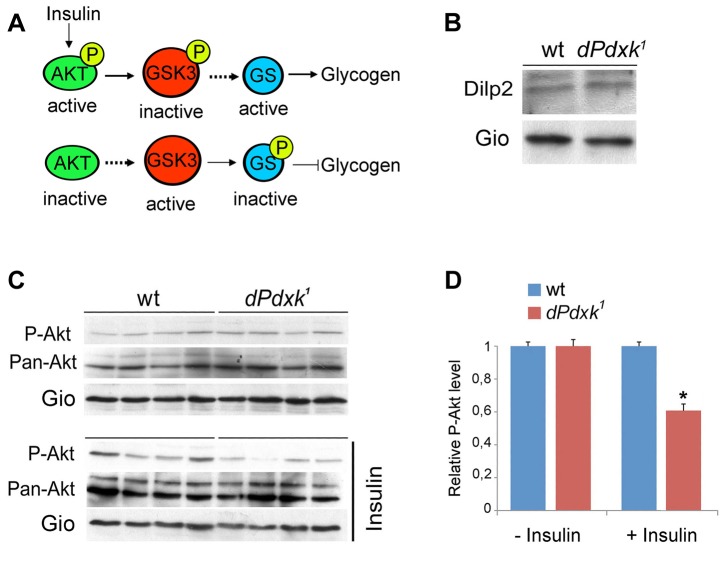
*dPdxk* mutants are defective in the insulin signaling pathway. (A) Simplified representation of the insulin-signaling pathway; see text for description. (B) Western blotting showing that *dPdxk^1^* mutants contain normal levels of DILP2. (C) Akt phosphorylation levels in wild type (wt) and *dPdxk^1^* mutant brains with or without insulin stimulation; P-Akt is the phosphorylated (Ser 505) Akt protein recognized by phospho-specific antibody; Pan-Akt is the is total Akt protein recognized by a specific antibody; Gio is a *Drososphila* Phosphatidylinositol transfer protein used as loading control (see Methods). (D) Quantification of the Akt phosphorylation levels. The intensity of the P-Akt bands was normalized to both total Akt and the Gio loading control. The columns are the means ±SE of 12 independent samples of 20 brains from 3 independent experiments (see Methods); the wild type pAkt/panAkt/Gio ratio has been arbitrarily set equal to 1. *, significantly different with p<0.05 in the Student t test. A short-exposure version of the blot from insulin-stimulated brains (Figure S2) provides visual evidence for a reduced P-Akt level in *dPdxk* mutants.

Studies on mammalian systems have shown that glucose accumulation within the cell might depend on either lack of insulin (type-1 diabetes) or defects in the insulin-signaling pathway (type-2 diabetes). Insulin promotes phosphorylation of AKT, which leads to phosphorylation and inactivation of glycogen synthase kinase 3 (GSK3) allowing glycogen formation (Figure 5A, top). In the absence of insulin, the active form of GSK3 phosphorylates and inhibits glycogen synthase (GS), the enzyme that catalyzes glycogen synthesis (Figure 5A, bottom) [34]. Thus loss of AKT or lack of AKT phosphorylation should result in glycogen synthesis inhibition and glucose accumulation.

Flies and mammalian systems employ similar mechanisms (Figure 5A) for regulation of carbohydrate homeostasis (reviewed in [35], [36]). The *Drosophila* genome encodes eight insulin-like peptides (DILPs) that are considered orthologous to mammalian insulin. These peptides are expressed in tissue- and stage-specific manner during development [37]–[39]. DILP2 is the closest homologue of human insulin and the most highly expressed DILP in the two bilaterally symmetric clusters of brain cells dubbed median neurosecretory cells (mNSCs) or insulin producing cells (IPCs); mNSCs/IPCs produce *Drosophila* insulin and are the main insulin suppliers during larval growth [37]–[39]. Using an antibody that specifically recognizes the mNSCs/IPCs [40] we found that *dPdxk^1^* mutant brains show a normal concentration of DILP-2 (Figure 5B). We next tested whether exogenous insulin has the ability of stimulating phosphorylation of Akt at Ser 505 in *dPdxk^1^* mutant brains; this residue is homologous to mammalian Ser 473 whose phosphorylation promotes full Akt activation [41] (Figure 5). After stimulation with human synthetic insulin, *dPdxk^1^* mutant brains displayed a limited but statistically significant reduction in the Akt phosphorylation level compared to controls (Figures 5C, D and S2). This suggests that glucose accumulation in *dPdxk^1^* mutants is at least in part due to a defect in insulin signaling.

### *dPdxk* mutants exhibit an increased uracil concentration in larvae but are only slightly sensitive to hydroxyurea

**Figure 6:**
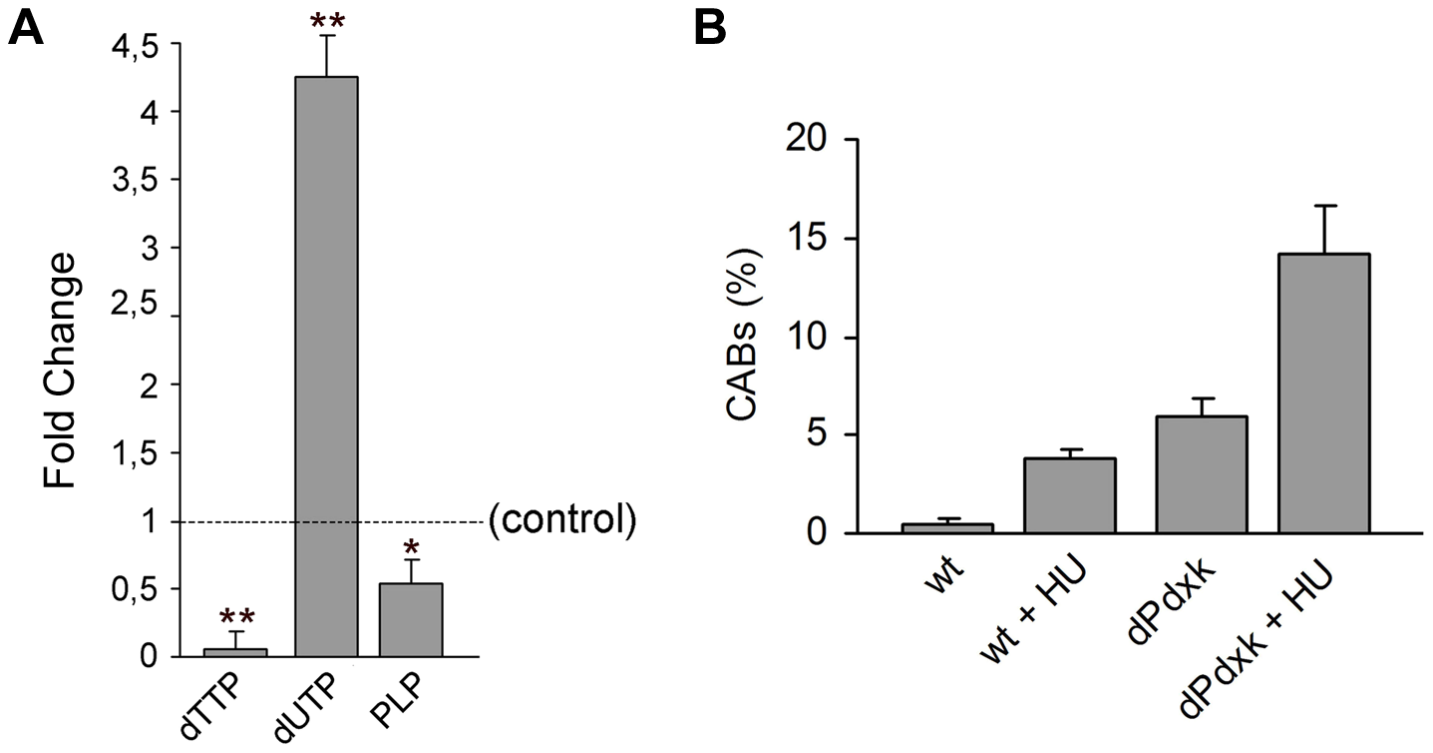
*dPdxk^1^* mutations cause nucleotide unbalance but are only weakly sensitive to hydroxyurea (HU). (A) *dPdxk^1^* mutant larvae have a very low dTTP content and accumulate uracil; they also exhibit a reduced PLP level. Values are the means of three independent experiments (±SD). * and **, indicate significant differences from wild type controls with p<0.05 and p<0.01, respectively. (B) *dPdxk^1^* mutants are weakly sensitive to HU. In HU-treated (2 mM) *dPdxk^1^* mutant brains, 1.6 % of metaphases exhibited shattered chromosomes; these metaphase were rarely observed (0.3%) in control and *dPdxk^1^* untreated mutant brains. Each bar in the graph represents the mean frequency of CABs (±SE) obtained by examining at least 600 metaphases form at least 6 brains.

PLP is cofactor of several enzymes involved in thymidylate (dTMP) biosynthesis. It has been previously shown that mutants in *BUD16*, the yeast gene that encode pyridoxal kinase, are defective in dTMP synthesis and incorporate more uracil nucleotides in their DNA than nonmutant controls [18]. Thus, in the attempt of defining the primary defect leading to CABs, we asked whether *dPdxk^1^* mutants accumulate uracil. We used HPLC/MS to determine uracil concentration in larval extracts and found that *dPdxk^1^* mutant larvae exhibit lower dTTP and higher dUTP levels compared with wild type controls (Figure 6A). HPLC/MS also showed that the PLP level in *dPdxk^1^* mutant larvae is approximately 50 % of that found in wild type controls (Figure 6A).

Because nucleotide unbalance can affect DNA synthesis and cause CABs [42] we asked whether brain cells of *dPdxk^1^* mutants were sensitive to hydroxyurea (HU). HU is expected to cause a DNA replication stress because inhibits ribonucleotide reductase and thus decreases the production of deoxyribonucleotides. We treated brains for 15 min with 2 mM HU and then placed them in saline for 2.5 hours before fixation. This treatment did not substantially reduce the mitotic index in control and mutant brains. HU-treated *dPdxk^1^* brains displayed a CAB frequency that was only slightly higher than the sum of the frequencies observed in HU-treated controls and in untreated *dPdxk^1^* mutants (Figure 6B). Thus, *dPdxk* mutations cause little or no increase in the sensitivity of *Drosophila* cells to HU.

### *dPdxk* mutations increase AGE formation

**Figure 7:**
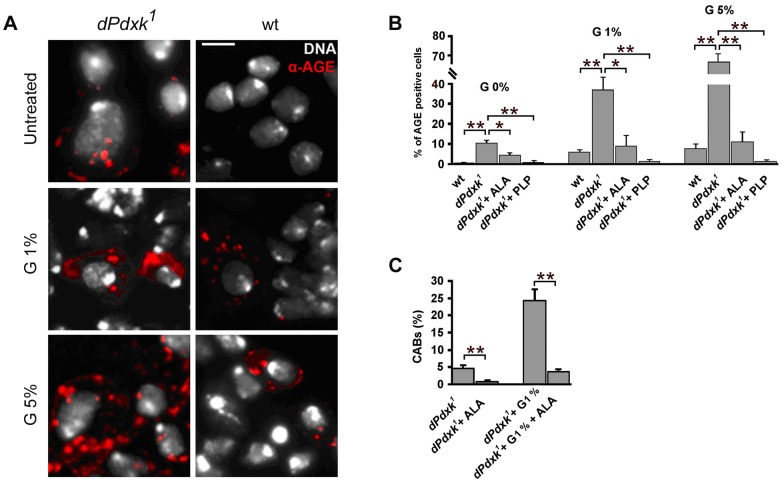
*dPdxk^1^* mutant brains exhibit higher frequencies of AGE-positive cells than wild type (wt) brains. (A) Examples of cells stained with a rabbit anti-human AGE antibody. Scale bar, 5 µm. (B) Frequencies of AGE-positive cells in wild type (wt) and *dPdxk* mutant brains exposed to different glucose (G) concentrations with or without ALA or PLP; bars represent the mean frequencies of AGE-positive cells (±SE) obtained by examining at least 300 cells/brain in 4 brains. At all glucose concentrations untreated *dPdxk^1^* mutant brains have a significantly higher frequency of AGE-positive cells than wild type controls and either ALA or PLP treated *dPdxk^1^* brains; (C) ALA reduces the frequency of CABs induced by *dPdxk^1^* or *dPdxk^1^* plus 1% G. Each bar in the graph represents the mean frequency of CABs (±SE) obtained by examining at least 600 metaphases form at least 6 brains. * and **, significantly different in the Student t test with p<0.03 and p<0.001, respectively.

It has been reported that PLP counteracts AGE (Advanced Glycation End products) formation [25], [26]. AGEs are heterogeneous molecules formed after nonenzymatic glycosylation (glycation) of proteins, lipids, or nucleic acids. AGE formation has been associated with the production of DNA damaging reactive oxygen species, and with the progression of several disorders including diabetes complications, neurodegenerative and cardiovascular diseases [43], [44]. Immunostaining with an anti-human AGE antibody revealed that both untreated and glucose-treated *dPdxk^1^* mutant brains (protocol 2; Figure 1B) consistently exhibit higher frequencies of AGE-positive cells than wild type controls. Here again, PLP addition prevented AGE formation (Figure 7A, B). We next treated *dPdxk^1^* brains with α-lipoic acid (ALA), an antioxidant compound that antagonizes AGE formation and cooperates with vitamin B6 in ameliorating insulin resistance in pre-diabetic rats [45], [46]. We incubated *dPdxk^1^* brains in 10 mM ALA, with or without glucose addition (protocol 2 Figure 1B). In all cases, ALA reduced the frequencies of AGE positive cells. Interestingly, ALA also reduced the frequency of CABs in both untreated and glucose-treated *dPdxk^1^* mutants (Figures 7B, C).

### Glucose causes CABs in PLP deficient human cells

**Figure 8:**
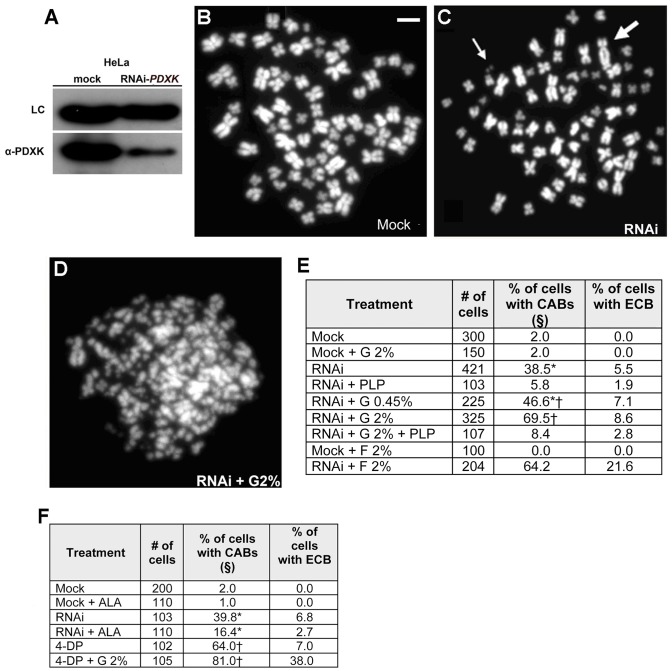
PLP deficiency causes CABs in HeLa cells. (A) Western blots showing that RNAi against *PDXK* in HeLa cells reduces the level of the PDXK protein to the 38% of the control level. (B–D) Examples of control and *PDXK* RNAi HeLa cells exposed to different glucose concentrations; the metaphase in panel D exhibits extensive chromosome breakage (ECB); the thin arrows point to chromosome fragments and the thick arrow to a dicentric chromosome. Scale bar, 10 µm. (E) CAB frequencies in mock-treated and *PDXK* RNAi (abbreviated with RNAi) cells incubated in media containing different glucose (G) or fructose (F) concentrations with or without PLP (2 mM); §, includes cells with ECB; * and †, significantly different in χ^2^ test with p<0.05 and p<0.001 respectively. (F) 4-DP (30 mM) induces CABs in HeLa cells, and this effect is potentiated by G; in contrast, ALA (10 mM) reduces the frequency of CABs induced by RNAi against *PDXK*. §, includes cells with ECB; * and †, significantly different in χ^2^ test with p<0.001 and p<0.01, respectively.

We finally asked whether glucose causes CABs in human cells with reduced PLP levels. RNAi-mediated PDXK depletion in HeLa cells (38 % of the control level; Figure 8A) resulted in a dramatic increase of CABs (Figures 8B–E). In addition, *PDXK* RNAi cells grown in media containing final glucose concentrations of 0.9 % or 2.45 % showed significant CAB increases compared to cells grown in standard medium (0.45 % glucose). Addition of PLP (at a final concentration of 2 mM) to either the standard or the 2.45 % glucose medium strongly reduced the CAB frequency (Figure 8E). Interestingly, *PDXK* RNAi cells exposed to high glucose concentrations displayed several metaphases with extensive chromosome fragmentation. These shattered metaphases (Figure 8D) were similar to the metaphases with multiple breaks observed in glucose-treated *Drosophila Pdxk* mutant brains (Figure 1A). The frequency of such metaphases was higher in fructose-treated cultures (2% fructose plus 0.45% glucose) than in cultures exposed to 2.45% glucose (Figure 8E). Thus, fructose appears to be more efficient than glucose in the induction of chromosome shattering in both *Drosophila* and human PLP-deficient cells.

We also found that HeLa cells behave as *Drosophila* brain cells in their responses to the vitamin B6 analog 4-DP and ALA. 4-DP caused extensive chromosome breakage in HeLa cells, which was potentiated by a high glucose concentration. Treatment of PDXK-depleted or 4-DP treated cells with ALA reduced the CAB frequency (Figure 8C).

## Discussion

Our results demonstrate that in organisms as distant as flies and humans sugars become potent clastogens when PLP is reduced. Previous studies showed that mutations in *BUD16*, the yeast gene encoding pyridoxal kinase, result in chromosome rearrangements, and that 4-DP induces DNA repair foci in human cells [18]. There is also evidence that vitamin B6 antagonizes AGE formation [19], [25], [26]. However, the deleterious genetic effects of sugars in vitamin B6 deficient cells have never been demonstrated.

### CAB formation and clastogenic effects of sugars *in dPdxk* mutant cells

Studies on *BUD16* yeast mutants showed that PLP is required for dTMP biosynthesis [18]. Cells bearing mutations in *BUD16* displayed excessive uracil incorporation into DNA compared to wild type, a condition that may lead to DSBs via uracil excision and production of abasic DNA sites. However, a deficiency of uracil glycosylase, the main enzyme that removes uracil from DNA, did not suppress the mutagenic effects of *BUD16*, indicating that uracil excision is not a major cause of DNA damage in cells with low PLP levels. Moreover, *BUD16* mutant cells showed an extreme sensitivity to hydroxyurea (HU), which inhibits ribonucleotide reductase that catalyzes the de novo synthesis of dNTPs. Based on these results, it has been suggested that PLP deficiency in yeast leads to DNA lesions and gross chromosomal rearrangements by causing a strong nucleotide imbalance that impairs DNA synthesis [18].

We have shown that *Drosophila* cells bearing mutations in *dPdxk* also exhibit a dUTP excess compared to wild type controls. However, *dPdxk* mutant cells do not appear to be particularly sensitive to HU. Untreated *dPdxk^1^* mutant cells and HU-treated wild type cells showed 6% and 4% CABs, respectively; HU-treated *dPdxk^1^* mutant cell displayed 16% CABs, a frequency that is only slightly higher than the frequency expected (10%) if HU and *dPdxk^1^* mutations acted independently. In contrast HU-treated brains of mutants in *tim2*, which encodes a replisome-associated factor, showed a ∼10-fold increase in the CAB frequency compared to the sum of the frequencies observed in untreated *tim2* mutant cells and HU-treated controls [47]. The finding that HU does not greatly exacerbate DNA damage in *Drosophila dPdxk* mutants suggests that the primary cause of CAB formation in these mutants is not nucleotide unbalance.

We have found that in both untreated and glucose-exposed *dPdxk* mutant brains there is a substantial increase in AGE formation. Most importantly we found that ALA reduces both AGE formation and the CAB frequency. Given that AGE formation is accompanied by the production of DNA damaging reactive oxygen species [43], [44] and that ALA is a potent antioxidant compound [46], we suggest that the DNA damage leading to CABs in *dPdxk* mutants, and especially in *dPdxk* mutants exposed to sugars, is at least in part a consequence of AGE formation. It is also possible that the lesions that cause CABs are generated by the simultaneous presence of AGE-linked reactive oxygen species and an unbalanced nucleotide pool. In this context, we propose that the higher frequency of CABs observed in fructose-treated brains compared to those exposed to glucose (Figure 1E) might reflect the higher efficiency of fructose in the initiation of the Maillard reaction that leads to AGE formation [48]–[50].

### Mutations in *dPdxk* lead to high intracellular glucose concentrations

Our results clearly show that the hemolymph and the brain cells of *dPdxk* mutants contain a higher glucose concentration than their wild type counterparts. We have also shown that *Pdxk* mutant brains incubated in glucose-enriched saline accumulate more glucose than wild type brains. Our results suggest that the increase in glucose concentration observed in *Pdxk* mutant brains is at least in part due to an acquired insulin resistance. It has been recently shown that *Drosophila* larvae reared on a high-sugar diet exhibit diminished insulin-induced Akt phosphorylation at Ser 505 compared to controls grown in normal medium [41]. However, the reduction in Akt phosphorylation observed in larvae grown in a sugar-rich medium [41] is substantially stronger than that found in *Pdxk* mutants. A possible reason for this difference in the response to insulin stimulation might be the time of exposure to high sugar. Larvae grown in sugar-rich medium were exposed to sugar throughout development. In contrast, it is likely that in *dPdxk* mutant larvae the maternal supply of Pdxk was progressively diluted during development becoming critically depleted only in late larval stages [51]. However, while this interpretation explains the differences between our data and those of Musselman and coworkers [41], it does not explain how *dPdxk* mutant acquired insulin resistance. We propose that PLP deficiency leads to an initial glucose accumulation through an as yet unknown mechanism and that this accumulation leads to insulin resistance. According to this interpretation, *dPdxk* mutant brains incubated in glucose- or fructose-enriched saline accumulate more sugar than wild type brains through two mechanisms, one linked to PLP deficiency per se and the other linked to insulin resistance.

### The mitotic spindles of *dPdxk* mutant are resistant to colchicine

The mitotic spindles of *dPdxk* mutant brains incubated in 1% glucose displayed an unexpected resistance to colchicine-induced depolymerization. This resistance was attenuated by treatment with tyrosine but not with glycine. Early work on human umbilical vein endothelial cells (HUVEC) has suggested a possible interpretation of these results. Culture of HUVECs in high glucose resulted in frequent loss of the terminal tyrosine residue from MTs, colchicine-resistant MTs and reduced cell proliferation [30], [31]. Colchicine resistance was attributed to tubulin detyrosination at MT ends, a reversible modification that renders MTs less dynamic and less resistant to nocodazole-induced depolymerization than tyrosinated MTs [28], [29]. Consistent with this interpretation La Selva and coworkers [31] showed that the defect in cell proliferation was corrected by tyrosine addition to the tissue culture medium. These authors did not attribute the loss of terminal tyrosine to tubulin glycation because they found that L glucose, which is not metabolized but can form Amadori bonds with protein amino groups, did not inhibit HUVEC proliferation.

There is abundant evidence that tubulin glycation can occur. For example in vitro glycation of rat brain tubulin increases with glucose concentration, and diabetic rats display a dramatic increase in glycosylated tubulin and defective tubulin polymerization [52]. Moreover recent work has shown that tyrosine glycosilation can occur also in humans [53]. Thus, although to the best of our knowledge there is no experimental evidence that tyrosine glycosylation can regulate MT dynamics, one can envisage that high intracellular glucose concentrations might cause tyrosine glycation. Under this assumption, the high glucose concentration in *dPdxk^1^* mutant cells would cause tyrosine glycation leading to detyrosinated MTs and colchicine resistance; addition of an excess of tyrosine would dilute the glycosylated tyrosine moiety, partially restoring the sensitivity of MTs to colchicine. Whatever the mechanism underlying the colchicine resistance of PLP depleted cells grown in high sugar, our finding has a potential translational impact and merits further study. An extrapolation of our results to human tumor therapy predicts that patients with vitamin B deficiency and hyperglycemia might be resistant to chemotherapy with MT-depolymerizing agents.

### The chromosome damage induced by PLP deficiency in HeLa cells is potentiated by sugar

Our experiments on HeLa cells have shown that the clastogenic effects of glucose and fructose in vitamin B6-deficient cells are evolutionarily conserved. HeLa cells in which the PDXK level was reduced to 38% of the control level by RNAi, displayed a 22-fold increase in the frequency of metaphases with CABs compared to non-RNAi cells. Both RNAi and non-RNAi cells were grown in standard DMEM medium, which contains 0.45% glucose and thus exceed the normal glucose concentration in blood, which is around 0.1 %. *PDXK* RNAi cells grown in DMEM containing final glucose concentrations of 0.9 % or 2.45 % (or 0.45 % glucose and 2% fructose) showed significant CAB increases compared to those grown in standard medium. Addition of PLP to either the standard or the 2.45% glucose medium strongly reduced the CAB frequency in *PDXK* RNAi cells. In addition, the PLP inhibitor 4-DP behaved as strong clastogen in HeLa cells, and its effect was potentiated by glucose addition to the medium. Together these results indicate that even moderate reductions in PLP level, such as that caused by a 62% diminution of PDXK, can results in a high CAB frequency. However, is possible that the elevated CAB frequency observed in *PDXK* RNAi cells is at least in part a consequence of the concomitant PLP deficiency and high glucose concentration (0.45%) in the standard DMEM medium used to grow HeLa cells. The central issue raised by these results is whether the clastogenic effects of sugars can occur in human patient with a vitamin B6 deficiency. We believe that this is quite possible, as deficiency of this vitamin can be caused by many dietary, genetic and pharmacological factors [54] and blood glucose level over 0.5% (500 mg/dL) have been observed in patients with hyperglycemic crises [55]. It is also conceivable that even relatively limited glycemia increases would cause CABs in people with particularly severe PLP deficiencies.

An interesting observation made on both *Drosophila* and human cells is that PLP deficiency accompanied by high sugar results in several metaphases with extensive chromosome fragmentation. The frequency of CABs in these metaphases is much higher than that expected from the Poisson distribution of CABs, suggesting that these CABs might result from the synergistic combination of two at least in part independent events such as PLP reduction and sugar increase. Given that PLP and sugar concentration can fluctuate we speculate that during interphase a small fraction of the cells that suffer PLP deficiency/high sugar can rapidly revert to relatively normal metabolic condition through either a PLP increase or a sugar decrease. This situation would results in chromosome shattering followed by DNA repair in a relatively normal cellular environment and might therefore give rise to cells with multiple and stable rearrangements such as those observed in chromothripsis [4]–[6], [56].

### PLP deficiency and human health

Inadequate intake of vitamin B6 has been associated with cancer risk [16], [57]–[59] and recent studies have shown that a high expression level of PDXK has a positive impact on survival of non-small cell lung cancer (NSCLC) patients [60]. In addition, growing evidence indicates that diabetes patients have a higher risk of various types of cancer [61]–[65]. Our findings provide an important link between these studies, suggesting that vitamin B6 deficiency accompanied by hyperglycemia might lead to chromosome damage and thus trigger carcinogenesis [1]–[3]. Our work further suggests that patients with hyperglycemia who also take drugs that antagonize PLP, should compensate by taking extra amounts of vitamin B6. Conversely, patients chronically treated with drugs that antagonize PLP should keep under control the level of sugar in their blood.

## Methods

### *Drosophila* strains

*dPdxk^1^* was isolated by a cytological screen of larval brain squashes from a collection of 1680 EMS-induced late lethals generated in Charles Zuker's laboratory (University of California, San Diego). *dPdxk^2^* [or *l(3)67Ab*], *Df(3L)29A6, Df(3L)AC1* and *Df(3L)ED4416* were all obtained from the Bloomington Stock center. The *dPdxk* mutations and the deficiencies were balanced over *TM6B* or *TM6C*, which carry the dominant larval marker *Tubby* (http://flybase.bio. indiana.edu/); homozygous and hemizygous mutant larvae were recognized for their non-*Tubby* phenotype. Germline transformation and complementation analysis are illustrated in Figure S1. All stocks were maintained on standard *Drosophila* medium at 25°C.

### Treatments of larvae and isolated brains

Flies were grown in standard *Drosophila* medium containing 4.5 % sucrose, and experiments were performed at 25 C°. To analyze the effects of *dPdxk* mutations and drug treatments we followed two different protocols (Figure 1B). In both protocols, wild type or *dPdxk* mutant larvae were grown in fly medium for 6 days with or without addition of sugars or drugs. In protocol 1, brains dissected from 6-day third instar larvae were incubated for 90 min in 2 ml of saline (0.7% NaCl) and 10^−5^ M colchicine and then fixed. In protocol 2, brains dissected from third instar larvae were incubated in 2 ml of saline supplemented with 10% fetal bovine serum (FBS, Gibco BRL) for 4 hours with or without addition of sugar or drugs and then fixed; 90 min before fixation colchicine (final concentration, 10^−5^ M) was added to the saline/FBS to collect metaphases. In the experiments with hydroxyurea (HU), we used a protocol different from those described in Figure 1B. Brains form six-day old third instar larvae were incubated for 15 min in saline with 2 mM HU (Sigma), washed, placed in saline for 2.5 h and then fixed; 1 h before fixation, brains were treated with 10^−5^M colchicine.

### Chromosome cytology and immunostaining

*Drosophila* metaphase chromosome preparations were obtained as previously described [66] and mounted in Vectashield H-1200 with DAPI (Vector Laboratories) to stain the chromosomes. Brain preparations for immunofluorescence and tubulin immunostaining were carried out according to Bonaccorsi et al. [67]. To stain the AGEs, brain squashes were incubated overnight at 4°C with a rabbit anti-human AGE antibody (1∶200 in PBS; ab23722, Abcam, UK), which was detected by a 1-hour incubation at room temperature with Alexa 555-conjugated goat anti-rabbit IgG (H+L) (1∶300 in PBS, Molecular Probes). Immunostained preparations were mounted in Vectashield medium H-1200 with DAPI. Observations were carried out using a Zeiss Axioplan fluorescence microscope equipped with CCD camera (Photometrics CoolSnap HQ).

### Glucose concentration and insulin pathway analysis

Glucose concentration in *Drosophila* hemolymph and brains was measured using the Infinity Glucose Hexokinase reagent (Thermo scientific). To measure glucose in the hemolymph, samples of 10 larvae were washed in NaCl 0.7%, 0.1% Triton X-100 and then in d-H_2_O. The hemolymph of these larvae was collected and its glucose content measured following the protocol of Rulifson et al. [38]. The values reported in Figure 2A are the means ±SE of 8 samples of 10 larvae. To measure glucose in brains, samples of 20 brains were placed in 40 µl of 10^−3^ M EDTA, 10^−2^M KH_2_PO_4_, and the complete protease inhibitor cocktail (Roche), mechanically homogenized and then centrifuged at 14,000 rpm for 10 min. The supernatant was collected with a micropipette and used for glucose measurement according to Rulifson et al. [38]. The measures reported in Figure 2B are the means ±SE of 4 samples of 20 brains.

Insulin stimulation of Akt phosphorylation was performed using recombinant human insulin (Sigma, I0516). Before stimulation, larvae were starved for 5 hours in an empty vial humidified with a drop of saline devoid of FBS. We then followed the protocol described by Musselman et al. [41]. However, at the end of the insulin stimulation procedure, instead of larvae, we homogenized samples of 20 isolated brains, which were then used for Western blotting analysis.

### Uracil and PLP quantitation

To quantitate nucleotides and PLP in larval extracts we used the HPLC/MS method described by D'alessandro et al. [68]. Briefly, third instar larvae (20 per sample) were washed in saline, homogenized, and then resuspended in 80 µl methanol; 100 µl of chloroform were then added to each tube. After 30 min mixing, 20 µl of ice-cold ultra-pure water was added to the tubes, which were centrifuged at 1,000 g for 1 min and then transferred to −20°C. After thawing, liquid phases were recovered and mixed to an equivalent volume of acetonitrile. The tubes were then centrifuged at 10,000 g for 10 min; the supernatants were recovered into 2 ml tubes, dried to obtain visible pellets, and resuspended in 200 µl of 5% formic acid in water. For metabolite separation we used an Ultimate 3000 high-resolution fast HPLC system (LC Packings, DIONEX, Sunnyvale, USA), with a Dionex Acclaim RSLC 120 C18 column “2.1 mm×150 mm, 2.2 µm”. A 0–95 % linear gradient of solvent A (0.1% formic acid in water) to B (0.1% formic acid in acetonitrile) was employed over 15 min followed by a solvent B hold of 2 min, returning to 100% A in 2 min and a 6 min post-time solvent A hold. ESI mass spectrometry was performed as described previously using a High Capacity ion Trap HCTplus (Bruker-Daltonik, Bremen, Germany) [68]. Validation of HPLC/MS-eluted metabolites was performed by comparison with the standard metabolites. ANOVA statistical analysis was carried out using GraphPad Prism 5 software.

### Western blotting and P-Akt measurement

Extracts for Western blotting of *Drosophila* proteins were prepared by lysing samples of 20 brains in 150 mM NaCl, 50 mM Tris-Hcl pH 7.5, 30 mM NaF, 25 mM b-glycerophosphate, 0.2 mM Na_3_VO_4_, Triton X-100 1%, and Complete protease inhibitor cocktail (Roche). Extracts were immunoblotted according to Somma et al. [69]; blotted proteins were detected using rabbit anti-DILP2 (1∶2000; a gift of E. Hafen), rabbit anti-Phospho (Ser 505)-*Drosophila* Akt (1∶1000; #4054, Cell Signaling), or rabbit anti-pan-Akt (1∶1000; #4691, Cell Signaling).

To determine the phosphorylation level of Akt we performed three different experiment similar to that shown in Figure 5C. We thus analyzed twelve 20-brain independent samples for wild type or *dPdxk* mutant third instar larvae. In each experiment we determined the intensities of P-Akt bands normalized to both total Akt and the loading control [Giotto (Gio) a *Drosophila* Phosphatidylinositol transfer protein; ref [70]. Measurements were performed on unsaturated bands using Image J software (http://rsb.info.nih.gov/ij/) for band quantification and normalization.

### Human cell procedures

HeLa cells were grown in DMEM (Gibco BRL) with 10% fetal bovine serum (FBS, Gibco BRL) in a humidified 5% CO_2_ atmosphere. *PDXK* siRNAs (SIHK1569, Sigma) were transfected using Lipofectamine 2000 (Invitrogen) according to the manufacturer's instructions. Mock-transfected and siRNA-transfected cells where grown for 24 hours in normal medium, which was then supplemented with glucose, fructose, PLP (2 mM), ALA (10 mM) or 4-DP (30 mM). In all cases, 72 hours after the beginning of treatments colcemid (0.05 µg/ml, Gibco BRL) was added to the cultures for 3 hours before fixation according to Revenkova et al. [71]. Chromosome preparations were mounted in Vectashield H-1200 with DAPI. Human cell extracts were prepared according to Cherubini et al. [72], and Western blotting was performed as described in Somma et al. [69]; PDXK was detected using a mouse anti-PDXK antibody (1∶500; 89006590, Abnova)

## Supporting Information

Figure S1 Mapping and functional characterization of the *dPdxk* gene. (A) Deficiency mapping of the *dPdxk^1^* mutations. The deficiencies that uncover the mutation are depicted in blue. (B) Constructs and vectors used for germline transformation and complementation analysis. The DNA fragment cloned into the pUAST vector [73] spans the promoter and the genomic region of the *dPdxk* gene (nucleotides 9352513-9354820); the other two constructs were cloned in a pCaSpeR-*tubulin* vector [74]. Germline transformation was carried out using standard methods. Complementation analysis was carried out using flies bearing the transgene on the second chromosome. *Tr/CyO; dPdxk^1^/TM6B* flies (*Tr* designates any homozygous viable transgene) were mated *inter se* to build *Tr/Tr; dPdxk^1^/TM6B* stocks. *Tr/Tr; dPdxk^1^/dPdxk^1^* animals from these stocks were then examined for viability and the presence of CABs in larval brains. All transgenes rescued the CAB phenotype of *dPdxk* mutants; the transgene placed under the control of the endogenous promoter also rescued the *dPdxk^1^* lethal phenotype.
(TIF)

Figure S2 Levels of phosphorylated Akt (P-Akt) in insulin-stimulated wild type (wt) and *dPdxk* mutant brains. The Western blot (WB) shown here is a short-exposure version of the WB of Figure 5, but it does not include Pan-Akt immunostaining, which was obtained after stripping the membrane stained for P-Akt and Giotto (Gio). Note that the P-Akt bands are more intensely stained in wild type than in *dPdxk* mutants.
(TIF)
