# Supplementary figures and images for: Sugar and Chromosome Stability: Clastogenic Effects of Sugars in Vitamin B6-Deficient Cells
Source: PLoS Genet. 2014 Mar 20;10(3):e1004199. doi: 10.1371/journal.pgen.1004199 (PMC3961173; doi:10.1371/journal.pgen.1004199)

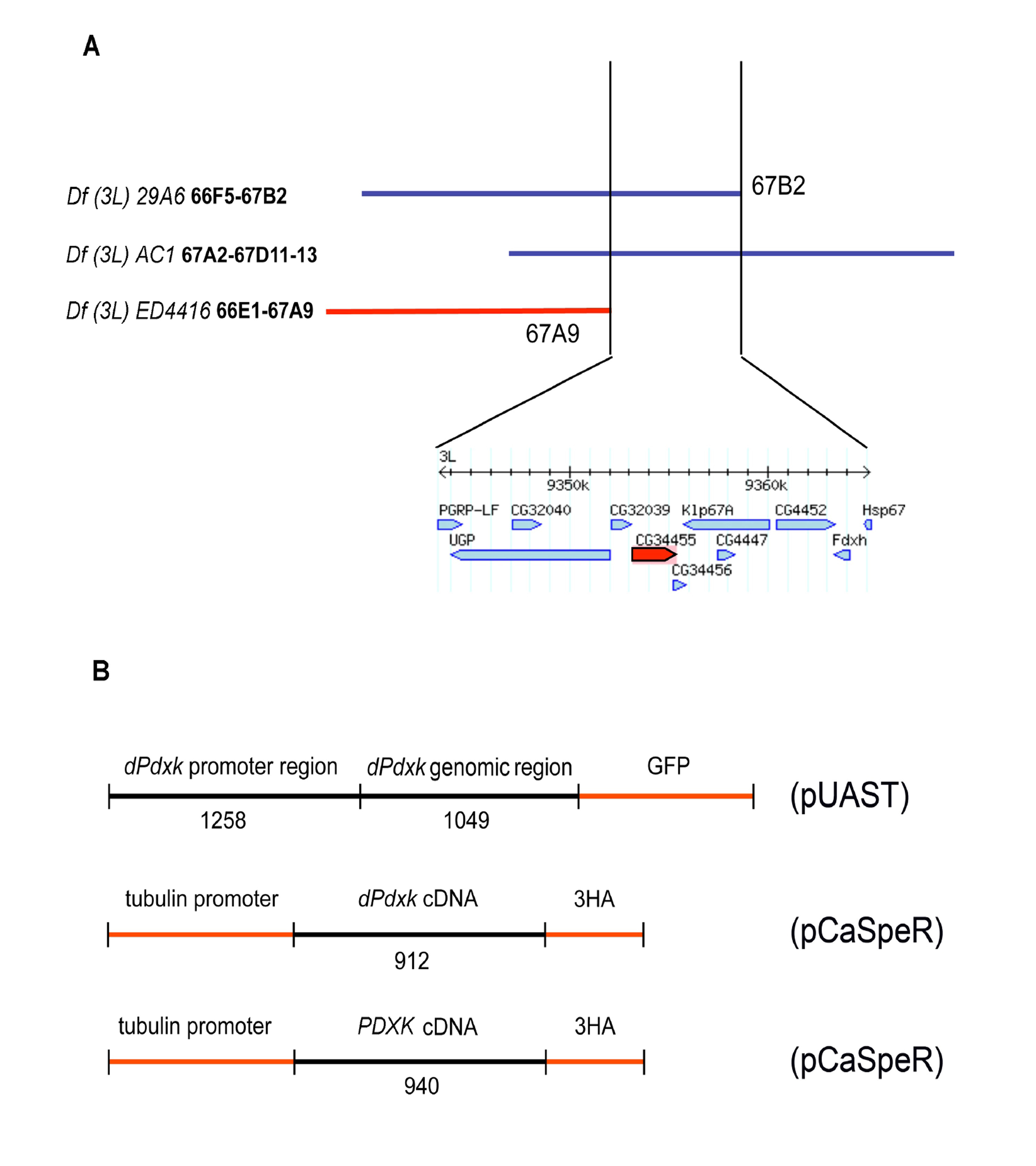

Supplement: Figure S1 — Mapping and functional characterization of the dPdxk gene. (A) Deficiency mapping of the dPdxk1 mutations. The deficiencies that uncover the mutation are depicted in blue. (B) Constructs and vectors used for germline transformation and complementation analysis. The DNA fragment cloned into the pUAST vector [73] spans the promoter and the genomic region of the dPdxk gene (nucleotides 9352513-9354820); the other two constructs were cloned in a pCaSpeR-tubulin vector [74]. Germline transformation was carried out using standard methods. Complementation analysis was carried out using flies bearing the transgene on the second chromosome. Tr/CyO; dPdxk1/TM6B flies (Tr designates any homozygous viable transgene) were mated inter se to build Tr/Tr; dPdxk1/TM6B stocks. Tr/Tr; dPdxk1/dPdxk1 animals from these stocks were then examined for viability and the presence of CABs in larval brains. All transgenes rescued the CAB phenotype of dPdxk mutants; the transgene placed under the control of the endogenous promoter also rescued the dPdxk1 lethal phenotype. (TIF) [file pgen.1004199.s001.tif]

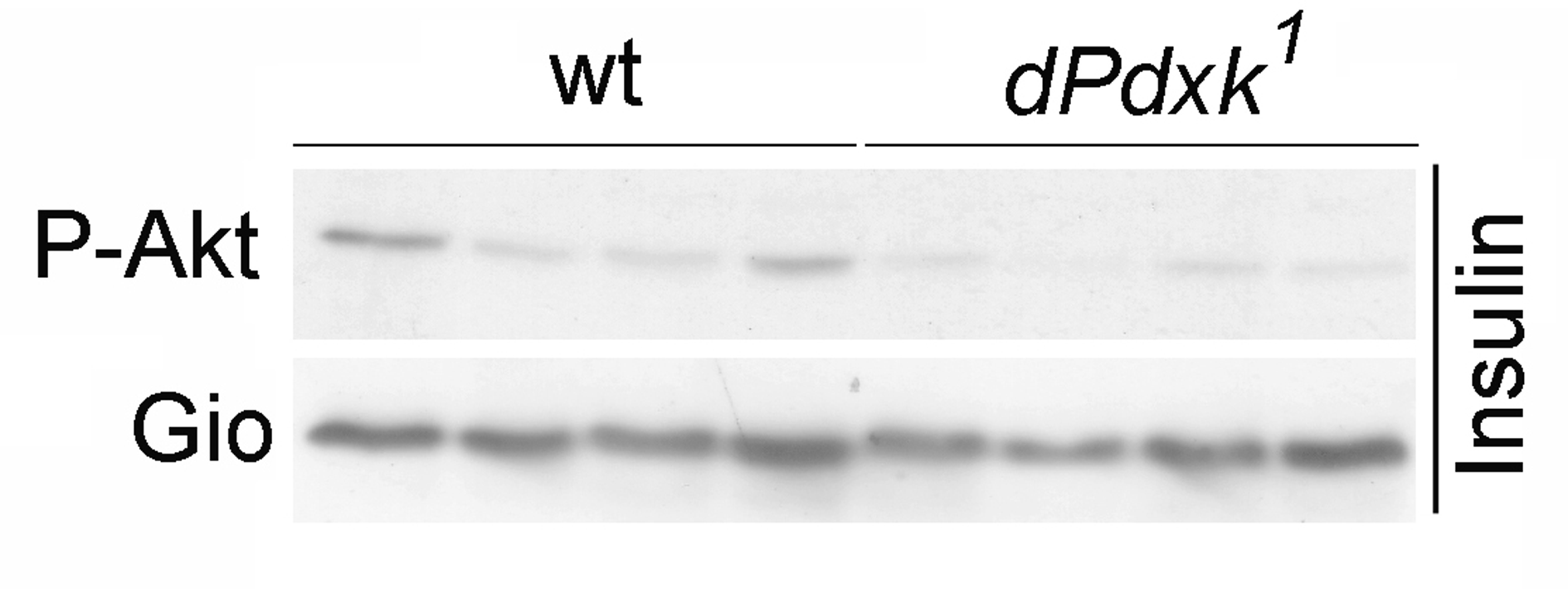

Supplement: Figure S2 — Levels of phosphorylated Akt (P-Akt) in insulin-stimulated wild type (wt) and dPdxk mutant brains. The Western blot (WB) shown here is a short-exposure version of the WB of Figure 5, but it does not include Pan-Akt immunostaining, which was obtained after stripping the membrane stained for P-Akt and Giotto (Gio). Note that the P-Akt bands are more intensely stained in wild type than in dPdxk mutants. (TIF) [file pgen.1004199.s002.tif]
